# Geographic and demographic disparities in sepsis and renal failure mortality: a CDC WONDER-based narrative review of 1999–2020 trends

**DOI:** 10.1097/MS9.0000000000004483

**Published:** 2025-12-04

**Authors:** Zaima Afzaal, Ayesha Khan, Tirath Patel, Saman Rauf, Fnu Kritika, Amna Zaman Khan, Ayman Irshad, Ali Hashim, Fatima Aslam, Hamza Sohail, Hamza Zaffar, Ayesha Khan, Salman Hassan, Ubaid Ahmed, Kehaan Rizvi

**Affiliations:** a Department of Medicine, Ameer-ud-Din Medical College, Lahore, Pakistan; b Department of Medicine, Dow University of Health Sciences, Karachi, Pakistan; c Department of Internal Medicine, Trinity Medical Sciences University School of Medicine, Ratho Mill, Kingstown, St Vincent, Saint Vincent and the Grenadines; d Department of Medicine, Fatima Jinnah Medical University, Lahore, Pakistan; e Department of Medicine, Indira Gandhi Institute of Medical Sciences, Patna, Bihar, India; f Department of Medicine, Al Aleem Medical College, Lahore, Pakistan; g Department of Medicine, Amna Inayat Medical College, Lahore, Pakistan; h Department of Medicine, Services Institute of Medical Sciences, Lahore, Pakistan; i Department of Medicine, Quaid-e-Azam medical college Bahawalpur, Pakistan; j Department of Medicine, Ziauddin Medical College, Karachi, Pakistan; k Department of Medicine, Nishtar Medical University, Multan, Pakistan; l Department of Medicine, Dow Medical College, Karachi, Pakistan; m Department of Medicine, Karachi Medical and Dental College, Karachi, Pakistan

**Keywords:** acute renal failure, CDC WONDER, mortality rates, sepsis

## Abstract

**Background:**

Sepsis and acute renal failure (ARF) are major contributors to mortality in critically ill patients in the United States. Despite advancements in critical care, trends in mortality associated with these conditions among adults aged ≥55 years have not been well studied.

**Methods:**

The Centers for Disease Control and Prevention Wide-Ranging Online Data for Epidemiologic Research (CDC WONDER) database was utilized to retrospectively analyze national mortality trends related to sepsis and ARF in the United States from 1999 to 2020, stratified by sex, race/ethnicity, age groups, U.S. Census region, and state.

**Results:**

The overall AAMR exponentially increased from 9.4 in 1999 to 18.5 in 2009 (APC: 7.73; 95% CI: 6.11 to 10.30) and it gradually increased to 22.6 in 2020 (APC: −0.14; 95% CI: −1.58 to 0.99, *P*-value = 0.819), with an average annual percentage (AAPC) of 3.53 (95% CI: 3.02 to 4.11, *P*-value = < 0.000001). Men had consistently higher AAMRs than women from 1999 (AAMR men: 12.4 vs. women: 7.5) to 2020 (AAMR men: 27.9 vs. women: 18.5), the AAPC for men is 3.22 (95% CI: 2.58 to 4.00) and for women is 3.96 (95% CI: 3.56 to 4.42). When stratified by race, the highest overall AAMR was observed in Black Americans (25.4%) and the lowest in Asians (13.4%). Overall, AAMR was highest for the South (19.5) and lowest for the Northeast (14).

**Conclusions:**

Following a period of dynamic fluctuations, sepsis and ARF-related mortality in U.S. adults have increased over the last 20 years. Focused interventions in prevention and treatment are crucial to mitigate the escalating impact of sepsis and ARF-related mortality.

## Introduction

Sepsis is defined as a disruption in the function of an organ due to an unregulated immune response to an infectious agent. Kidneys are among the most commonly failing organs in sepsis, with a high-mortality rate related to acute kidney injury (AKI), clinically characterized by oliguria, rising serum creatinine, and reduced glomerular filtration. It is one of the most frequent and serious complications of sepsis. The coexistence of sepsis and AKI is associated with prolonged hospital stays, higher healthcare costs, and significantly increased mortality. In the United States (US), AKI prevalence in sepsis inpatients rose from 39.1% in 2010 to 41% in 2019^[1]^. Among patients with sepsis, AKI was included as a contributing cause in 38.2% deaths in seven states of the United States^[2]^.

Incidents and mortalities related to sepsis increase as the U.S. population ages. Previous data suggest that sepsis and AKI-related mortalities have regional, age and gender-specific variations, primarily due to differences in underlying diseases and infection sites^[3]^.

Identifying demographic, regional, age and gender-specific distribution of mortality associated with AKI can help identify high-risk populations and can help in timely targeted intervention. Therefore, it is of utmost importance to evaluate demographic, regional, age and gender-specific differences in sepsis associated with AKI-related mortality from 1999 to 2019 among adults in the United States.

This manuscript is made compliant with the TITAN checklist to ensure transparency in the reporting of Artificial Intelligence^[4]^. Although advances in sepsis recognition and management have improved outcomes in some settings, disparities in mortality persist. Population-based data are critical to identify at-risk groups and inform resource allocation. This study evaluates long-term national mortality trends in sepsis with AKI among U.S. adults aged ≥55 years, stratified by sex, race, region, and urbanization.

## Methods:

### Study design

The data for this descriptive analysis were retrieved using the Centers for Disease Control and Prevention Wide-Ranging Online Data for Epidemiologic Research (CDC WONDER) Database^[5]^. The International Classification of Diseases (ICD-10) codes N17 for Acute Renal Failure (ARF) and A40 and A41 for sepsis were used to extract data from death certificates^[6]^. Studies on patients with ARF^[7]^ and sepsis^[8]^ have already used this code. This analysis included all individuals aged ≥55 years who had acute renal failure and sepsis listed anywhere on their death certificate in the United States between 1999 and 2020^[9]^. Data were retrieved from the multiple cause of death dataset. As CDC WONDER provides de-identified, publicly available data, Institutional Review Board (IRB) approval was not required. The guidelines from the Strengthening the Reporting of Observational Studies in Epidemiology (STROBE) study were followed in this study^[10]^.

### Data abstraction

Mortality data related to ARF and sepsis for individuals aged 55 years and older in the United States was collected from 1999 to 2022. Data were stratified by year, gender, census region, and urban–rural classification. Census regions – Northeast, Midwest, South, and West – were defined per the U.S. Census Bureau^[5]^. Urban and rural areas were classified based on the 2013 National Center for Health Statistics’ Urban–Rural Classification Scheme for Counties^[11]^. Areas with populations ≥50 000 were considered urban, while those with populations <50 000 were categorized as rural. Counties were further divided into six levels of urbanization: four metropolitan categories (large central metro, large fringe metro, medium metro, and small metro) were classified as urban, while two nonmetropolitan categories (micropolitan and noncore) were designated as rural. Crude death rates per 100 000 persons were extracted with corresponding 95% confidence intervals (CI), along with age-adjusted mortality rates (AAMR) standardized to the 2020 U.S. population.

### Statistical analysis

Temporal trends in AAMR were evaluated using joinpoint regression to identify significant changes over time. To examine the trends of sepsis and ARF-related mortality, the Joinpoint Regression Program (Version 5.3.0.0) was used^[12]^. This method fits linear segments on a logarithmic scale and detects “joinpoints” where trends shift^[13]^. As our research spans over 22 years, the software was configured to identify up to four joinpoints indicating significant trend changes. When trend variations were more distinct, fewer joinpoints were identified. Therefore, the analysis allowed for the detection of between 0 and 4 joinpoints. The Grid Search method (2, 2, 0), combined with empirical quantile and weighted Bayesian Information Criterion (BIC) methods, was employed to determine the optimal number of join points. Annual percent changes (APCs) and their corresponding 95% CIs were calculated for each segment, while the average annual percent change (AAPC) summarized the overall trend during the study period. An APC was deemed statistically significant if its 95% CIs excluded zero, with significance determined by two-tailed hypothesis testing at a threshold of *P* ≤ 0.05.

**HIGHLIGHTS**

- From 1999 to 2020, age-adjusted death rates due to sepsis and renal failure increased.
- Throughout all years, males consistently exhibited higher death rates than females.
- Among all the racial groups examined, Black Americans faced the highest mortality rates.
- The Southern and nonmetropolitan areas of the United States reported the highest rates of mortality.
- Despite overall advancements, disparities persisted based on geography, race, and urban versus rural settings.

## Results

### Gender:

**Figure 1.**
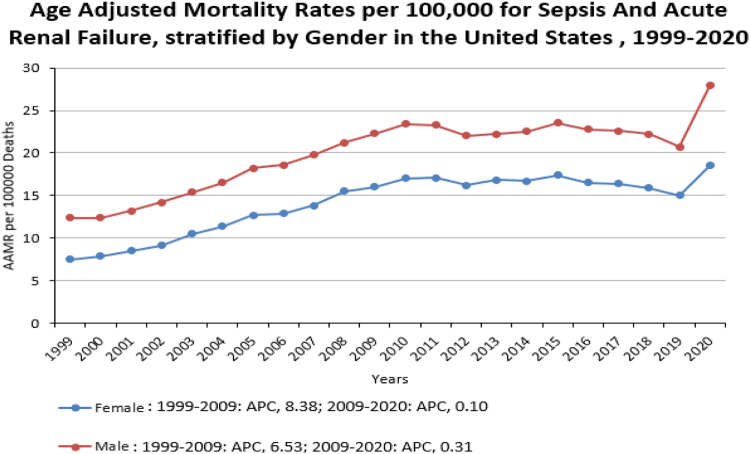
Age Adjusted Mortality Rates per 100,000 for sepsis and acute renal failure, stratified by Gender in the US, 1999-2020.

Throughout the study period, males consistently demonstrated a higher AAMR compared to females (overall AAMR for men: 20.5; 95% CI: 20.4–20.6; for women: 14.4; 95% CI: 14.3–14.5). In 1999, the AAMR for men was 12.4 (95% CI: 11.9–12.9), which increased to 22.3 in 2009 (APC: 6.53; 95% CI: 4.5–8.58), followed by an increase to 27.9; 95% CI: 27.4–28.4 in 2020 (APC: 0.31; 95% CI: −0.94 to 1.58). Correspondingly, the AAMR for women in 1999 was 7.5 (95% CI: 7.2–7.8), which increased to 16 in 2009 (APC: 8.39; 95% CI: 7.12–9.67), followed by a modest increase to 18.5; 95% CI 18.1–18.9 in 2020 (APC: 0.10; 95% CI: −0.76 to 0.97). (Fig. 1) (Table 2)

.

### Census region:

Throughout study, the highest AAMR was noted in Southern region at 19.5 (95% CI: 19.4–19.6), followed by the Western region at 16.8 (95% CI: 16.6–16.9), the Midwestern region at 15.3 (95% CI: 15.2–15.4), and the Northeastern region at 14 (95% CI: 13.9–14.1). In the Southern region, AAMR markedly increased from 1999 to 2009 (APC: 8.02; 95% CI: 5.9–10.2), followed by a slight growth till 2020 (APC: 0.50; 95% CI: −0.73 to 1.76). In the western region, AAMR increased from 1999 to 2009 (APC: 9.45; 95% CI: 7.3–11.6), followed by a minor rise from 2009 to 2020 (APC: 1.14; 95% CI: 0.09–2.2). In the Midwestern region, AAMR substantially increased from 1999 to 2008 (APC: 8.31; 95% CI: 6.31–10.34), followed by a slight rise from 2008 to 2020 (APC: 0.57; 95% CI: −0.35 to 1.49). In the Northeastern region, AAMR increased from 1999 to 2010 (APC: 4.70; 95% CI: 4.03–5.37), followed by a significant decline until 2018 (APC: −3.64; 95% CI: −4.69−2.58), and finally a sharp increase until 2020 (APC: 11.65; 95% CI: 2.41–21.73). (Table 1)

**Table 1:** Annual percentage changes (APCs) in sepsis and acute renal failure-related mortality trends in the United States, 1999–2020

Year interval	APC (95% CI)	*P*-value
Overall		
1999–2009	7.7330* (5.9407 to 9.5556)	<0.000001
2009–2020	−0.1434 (−1.2691 to 0.9952)	0.792649
Male		
1999–2009	6.5254* (4.7626 to 11.2910)	<0.000001
2009–2020	0.3138 (−1.8412 to 1.5758)	0.562687
Female
1999–2009	8.3876* (7.2518 to 9.8198)	<0.000001
2009–2020	0.1011 (−0.8362 to 0.8942)	0.787842
NH American Indian or Alaska Native		
1999–2010	8.4773* (5.2735 to 24.3912)	<0.000001
2010–2020	0.0677 (−5.2270 to 2.6206)	0.979404
NH Asian or Pacific Islander		
1999–2010	6.5921* (4.8699 to 9.2216)	0.001200
2010–2018	−2.7280* (−8.5505 to −1.0575)	0.008798
2018–2020	14.3283* (3.4344 to 21.3225)	0.004799
NH Black or African American		
1999–2008	5.8294* (3.9303 to 8.6417)	0.002000
2008–2018	−2.5871* (−6.3151 to −1.3964)	0.003599
2018–2020	17.0387* (4.9066 to 24.1104)	0.003999
NH White		
1999–2009	8.1062* (6.8919 to 9.6325)	<0.000001
2009–2020	0.6520 (−0.4047 to 1.5351)	0.179164
Hispanic or Latino		
1999–2009	6.7718* (3.6751 to 49.1589)	0.029594
2009–2018	0.5431 (−11.6618 to 5.0658)	0.994601
2018–2020	21.2130* (3.2122 to 36.3001)	<0.000001
WESTERN REGION		
1999–2009	9.4454* (7.6486 to 12.3936)	<0.000001
2009–2020	1.1385* (0.0537 to 2.1915)	0.043191
MIDWESTERN REGION		
1999–2008	8.3105* (6.6093 to 10.8428)	<0.000001
2008–2020	0.5662 (−0.4562 to 1.4088)	0.217157
NORTHEASTERN REGION
1999–2010	4.7031* (3.8012 to 5.8550)	0.000400
2010–2018	−3.6380* (−6.5917 to −2.4914)	0.002400
2018–2020	11.6490* (2.5165 to 16.8071)	0.010798
SOUTHERN REGION		
1999–2009	8.0216* (6.1827 to 11.8237)	<0.000001
2009–2020	0.5021 (−1.1463 to 1.7381)	0.392322
LARGE CENTRAL METRO		
1999–2009	6.2179* (4.1337 to 8.9328)	0.011598
2009–2018	−1.0233 (−6.3192 to 6.5628)	0.141572
2018–2020	9.7485* (0.0260 to 15.5241)	0.048790
LARGE FRINGE METRO
1999–2008	7.2118* (5.5857 to 9.7730)	<0.000001
2008–2020	−0.3404 (−1.2633 to 0.3838)	0.347131
MEDIUM METRO		
1999–2009	9.3547* (4.9397 to 23.8732)	0.011598
2009–2018	−0.5972 (−5.4403 to 14.0967)	0.354329
2018–2020	7.3880 (−0.2048 to 12.1709)	0.069586
SMALL METRO		
1999–2011	8.6034* (6.9629 to 11.8559)	<0.000001
2011–2020	0.4968 (−2.0077 to 2.2397)	0.525895
MICROPOLITAN (NONMETRO)		
1999–2011	8.3796* (5.4363 to 16.0357)	0.001600
2011–2015	1.7697* (0.2614 to 10.5968)	0.026795
2015–2018	−3.9872* (−6.8905 to 0.5239)	0.015197
2018–2020	11.3659* (5.6409 to 16.3528)	0.002000
NONCORE (METRO)		
1999–2009	7.8029* (6.1511 to 11.6491)	<0.000001
2009–2020	2.6156* (0.8637 to 3.7795)	0.019196

Statistically significant *P-*values are highlighted bold.

APC, annual percent change; CI, confidence interval

**Table 2:** Characteristics of sepsis and acute renal failure (ARF) disease-related deaths among individuals in the United States between 1999 and 2020

Variable	Deaths (%)	AAMR (95% CI)
Acute Renal Failure (ARF) and Sepsis	269 990(100%)	16.9 (16.8–17)
Sex		
Male	134 910 (49.97)	20.5 (20.4–20.6)
Female	135 080 (50.03%)	14.4 (14.3–14.5)
Race		
NH American Indian or Alaska Native	1923 (0.70%)	17.9 (17.1–18.8)
NH Asian or Pacific Islander	7732 (2.90%)	13.4 (13.1–13.7)
NH Black or African American	36 356 (13.5%)	25.4 (25.2–25.7)
NH White	223 979 (83.0%)	16.2 (16.1–16.2)
Hispanic or Latino	20 374 (7.5%)	18.4 (18.1–18.6)
Census Region		
North East	44 367 (16.4%)	14 (13.9–14.1)
Midwest	55 481 (20.50%)	15.3 (15.2–15.4)
South	112 960 (41.8%)	19.5 (19.4–19.6)
West	57 182 (20.2%)	16.8 (16.6–16.9)
Urbanization		
Large Central Metro	75 562 (28.0%)	17.3 (17.2–17.5)
Large Fringe Metro	60 267 (22.3%)	15.9 (15.8–16.1)
Medium metro	58 180 (21.5%)	17.1 (16.9–17.2)
Small metro	26 390 (9.8%)	16.7 (16.5–16.9)
Micropolitan (non-metro)	27 979 (10.4%)	17.6 (17.4–17.8)
Noncore (non-metro)	21 612 (8%)	17.1 (16.9–17.4)

.

### Race:

When analyzed according to race/ethnicity, AAMRs were highest among NH Black or African American patients, followed by Hispanic or Latino, NH American Indian or Alaska Native, NH White, and NH Asian or Pacific Islander populations (overall AAMR NH Black or African American: 25.4, 95% CI 25.3–25.7; Hispanic or Latino: 18.4, 95% CI 18.1–18.6; NH American Indian or Alaska Native: 17.9; 95% CI 17.1–18.8; NH White 16.2, 95% CI 16.1–16.2; NH Asian or Pacific Islander: 13.4, 95% CI 13.1–13.7). In brief, the AAMR for Hispanics increased from 1999 to 2020, while those for NH Whites increased sharply from 1999 to 2009 and then gradually increased until 2020. The AAMR for NH American Indians or Alaska Natives increased from 1999 to 2010 and then remained steady until 2020. The AAMR for NH Asian or Pacific Islander spikes from 1999 to 2010 (APC: 6.6, 95% CI: 5.1–8.1), followed by a sharp decline from 2010 t 2018 (APC: −2.7, 95% CI: −4.4 to 0.9) and finally again a steep increase from 2018 to 2020 (APC: 14.3, 95% CI: 2.8–27.1). The AAMR for NH Black or African American also increased sharply from 1999 to 2008, then decreased until 2018, and subsequently increased again until 2020. (Fig. 3, Table 1, Table 2).

### Urbanization:

**Figure 2.**
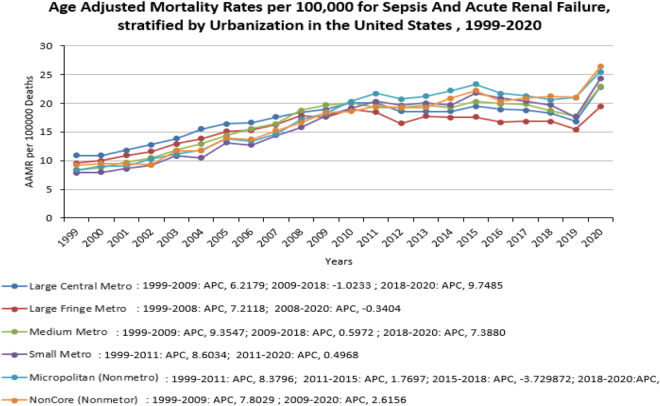
Age Adjusted Mortality Rates per 100,000 for sepsis and acute renal failure, stratified by Urbanization in the US, 1999-2020.

Upon stratification by urbanization, AAMRS were highest among Micropolitan (Non-Metro) areas, followed by Large Central Metro, Non-core (Non-Metro), Medium Metro, Small Metro, and Large Fringe Metro areas. [Overall AAMR Micropolitan (Non-Metro) 17.6, 95% CI 17.4–17.8; Large Central Metro 17.3, 95% CI 17.2–17.5; Non-core (Non metro) 17.1 95% CI 16.9–17.4; Medium Metro 17.1 95% CI 16.9–17.2; Small Metro 16.7 95% CI 16.5–16.9; large fringe metro 15.9 95% CI 15.8–16.1]. The AAMRs for Large Central Metro, Medium Metro, and Small Metro increased rapidly at first and then increased steadily till 2020. There was a sharp increase in the AAMR for large fringe metro areas from 1999 to 2008 (APC: 7.2, 95% CI: 5.1–9.3), after which it decreased slowly until 2020 (APC: −0.3, 95% CI: −1.2 to 0.5). The AAMR for Micropolitan increased sharply from 1999 to 2011 (APC: 8.7, 95% CI, 7.7–9.7), then became almost steady from 2011 to 2018 (APC: −0.3, 95% CI −2.0 to 1.44), and then took a marked increased from 2018 to 2020 (APC: 7.9, 95% CI −1.8 to 18.7). The AAMR for Non-core (non-metro) increased sharply from 1999 to 2009 (APC: 7.8,95% CI 5.8–9.8) and then increased from 2009 to 2020 at a relatively lower rate than before (APC: 2.6, 95% CI 1.4–3.8) (Fig. 2, Table 1)

**Figure 3.**
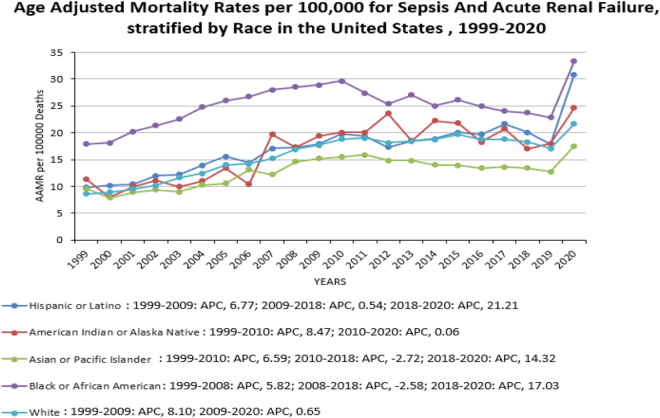
Age Adjusted Mortality Rates per 100,000 for sepsis and acute renal failure, stratified by Race in the US, 1999-2020.

.

### Overall:

**Figure 4.**
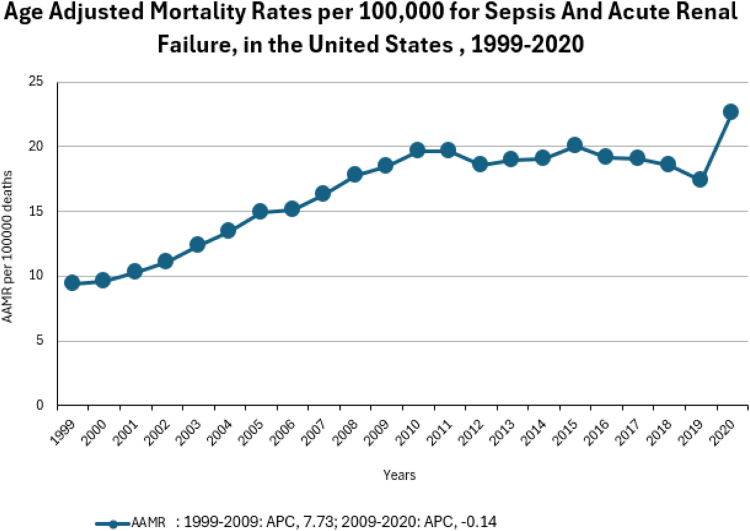
Age Adjusted Mortality Rates per 100,000 for sepsis and acute renal failure in the US, 1999-2020.

The AAMR for septicemia and ARF-related deaths was 9.4 in 1999 and 22.6 in 2020. The overall AAMR increased from 1999 to 2009 (APC: 7.7; 95% CI 5.9–9.5), followed by a slight decrease from 2009 to 2020 (APC −0.1; 95% CI −1.2 to 0.9). (Fig. 4)

.

## Discussion

This study highlights the mortality burden associated with sepsis and ARF, revealing significant trends and contributing factors influencing patient outcomes. The mortality rates varied significantly among different racial groups, with NH Black or African American patients having overall AAMR as high as 25.4 per 100 000, and NH Asian or Pacific Island patients with low rates (13.4 per 100 000). Although the AAMR of the NH Black population saw a decline from 2008 to 2018, all the races saw an increasing trend of mortality from 2018 to 2020. Our study examined temporal trends in mortality associated with sepsis and ARF, highlighting significant shifts in clinical outcomes over time. The finding suggests an overall improvement in survival rates, likely due to advancements in early diagnosis, treatment strategies, and critical care management^[14]^.

Despite advancements in medical care, sepsis and ARF continue to be associated with raised mortality rates, especially in critically ill patients. Several factors, including early recognition, timely intervention, comorbid conditions, and healthcare disparities, play crucial roles in determining patient survival^[15]^. The widespread adoption of sepsis bundles, early goal-directed therapy, better antimicrobial stewardship, and advancements in renal replacement therapy (RRT) have contributed to better outcomes^[16,17]^. However, mortality remains substantial, particularly in patients who develop septic shock or severe kidney injury requiring dialysis.

The combination of sepsis and ARF creates a vicious cycle, where kidney dysfunction exacerbates sepsis-related organ failure, further increasing mortality risk^[17]^. Delays in initiating appropriate resuscitation, antibiotic therapy, and renal support have been linked to higher mortality rates. The use of electronic health records, sepsis screening tools, and biomarkers has improved early detection^[15]^. However, variations in adherence to sepsis protocols across healthcare facilities may contribute to differences in patient outcomes. These shared factors highlight the bidirectional relationship, as sepsis often triggers renal injury while ARF worsens sepsis progression through immune and metabolic dysfunction, leading to multi-organ failure.

Sepsis and ARF share overlapping risk factors, with advanced age and comorbidities such as diabetes, hypertension, cardiovascular disease, chronic kidney disease, and malignancy increasing susceptibility through impaired immunity and reduced renal resilience. Hospital exposures – including critical illness, surgery, trauma, intensive care admission, and nephrotoxic agents – further heighten vulnerability to renal injury in septic patients. Pathophysiologically, hemodynamic instability and septic shock cause renal hypoperfusion and ischemic damage, while systemic inflammatory responses mediated by cytokines and microvascular dysfunction directly injure renal tissue.

The COVID-19 pandemic starkly illuminated these intersections. Numerous studies showed that patients with pre-existing kidney injury were at significantly higher risk of severe COVID-19, ICU admission, and death. This vulnerability stemmed not only from poorly controlled baseline kidney function but also from heightened inflammatory and dysregulated immune responses characteristic of hypertensive individuals, potentially mediated by endothelial dysfunction and alterations in angiotensin-converting enzyme 2 (ACE2) expression^[18,19]^.

The statistics showed men bearing higher AAMRs than women throughout the study period. There was a period of upsurge in mortality rates from 1999 to 2020. This pattern was uniform primarily among both older men and women. Differences in health-seeking behaviors and treatment patterns may also contribute, with men presenting later in the disease course, leading to delayed interventions. Women often encounter the healthcare system through gynecologic or contraceptive visits, increasing opportunities for blood pressure screening. In contrast, gender norms, where men are expected to be stoic and avoid seeking help, result in reduced utilisation of preventive care and delayed detection of hypertension or other comorbidities^[20]^. Biologically and behaviourally, men also bear a higher burden of risk factors that compound sepsis outcomes, as men have an earlier onset of cardiovascular diseases and are more prone to conditions like chronic obstructive pulmonary disease, which is a common comorbidity impacting sepsis prognosis^[21,22]^. Black and Hispanic populations face disproportionately higher rates of sepsis and ARF due to structural inequities, comorbidities, lifestyle risks, and limited healthcare access, particularly in rural areas^[23]^.

Upon stratification based on urbanization, the Micropolitan (non-metro) population showed higher mortality rates that points to limited access to advanced sepsis management, targeted therapies and renal replacement therapy may contribute to poorer outcomes^[24]^. All sectors of society experienced an initial steep rise in mortality rates till 2010 and remained almost steady until 2020, with few variations during this period (Table 2). In rural areas, greater distances to hospitals often delay critical care, leading to fewer referrals and admissions for severe conditions, such as sepsis and hypertensive crises. Conversely, urban residents, particularly in affluent regions, benefit from better healthcare access and more frequent referrals, largely due to socioeconomic advantages^[25]^. This disparity reflects the growing rural mortality penalty, where low-income rural populations face shorter lifespans and higher all-cause mortality compared to urban peers^[26]^. Social determinants, including poverty, education, and health behaviors, exacerbate these outcomes. Rural communities also experience higher rates of preventable hospitalizations, delayed cancer screenings, and increased mortality from cardiovascular disease, infant deaths, and COVID-19^[26,27]^. Meanwhile, rising hospice use marks a shift in end-of-life care^[28]^.

The study aimed to determine how mortality rates associated with sepsis and ARF varied across different geographical sectors. Some areas, such as the Southern region, exhibited high mortality rates, while the Northwestern region had the lowest mortality rate among all regions. In the Midwestern region, AAMR markedly increased in the initial period of the study, then stabilised from 2008 to 2020. These findings emphasize the significance of location-specific factors, such as healthcare infrastructure, resource availability, and variations in clinical practice, in the development of new treatment methods^[24]^.

Sepsis and ARF are pathophysiologically intertwined, with numerous overlapping risk factors that predispose patients to both conditions. Advanced age is a major determinant, as reduced renal reserve, immunosenescence, and the burden of chronic illness increase susceptibility. Comorbidities such as diabetes mellitus, hypertension, cardiovascular disease, chronic kidney disease, and malignancy further amplify vulnerability through mechanisms of impaired host defence, endothelial dysfunction, and reduced renal resilience.

Hospital-related factors, including critical illness, major surgery, trauma, and intensive care admission, contribute to the high incidence of sepsis and ARF, while exposure to nephrotoxic agents – such as aminoglycosides, vancomycin, NSAIDs, and iodinated contrast – exacerbates renal injury in septic patients. Hemodynamic instability and septic shock result in renal hypoperfusion and ischemic tubular damage, whereas the systemic inflammatory cascade of sepsis, mediated by cytokines and microvascular dysfunction, directly injures renal tissue.

Socio-demographic disparities also play an important role. Black and Hispanic populations in the United States experience disproportionately higher rates of both sepsis and ARF, reflecting structural inequities, comorbidity burden, and barriers to timely care. Lifestyle factors, including smoking, obesity, and alcohol use, further increase risk, while delayed access to healthcare in rural areas exacerbates the likelihood of sepsis-induced ARF.

Collectively, these shared risk factors underscore the close clinical and epidemiologic relationship between sepsis and ARF. Not only does sepsis frequently precipitate renal injury, but ARF also worsens the course of sepsis by impairing immune function, disrupting metabolic homeostasis, and contributing to multi-organ failure.

These findings have helped identify the high-risk populations and communities in the United States. A few recommended strategies to better apply these findings for the betterment of national health include regional sepsis awareness campaigns. Inauguration of mobile dialysis units in non-metro areas is also an important measure that can be taken. Targeted interventions and new policies focused on these sectors of society can help mitigate the risk of this disease and reduce the burden on the U.S. healthcare system.

## Limitations

There are some noteworthy limitations that need to be pointed out. This study relies on ICD-10 coding, which may have evolved over time. Inability to assess the temporal sequence of sepsis vs AKI is another important limitation. Furthermore, the absence of clinical variables (labs, comorbidities, treatment) proves challenging in taking into account the treatment regimen of patients. The lack of data on baseline comorbidities of individuals, the lack of evaluation of confounding factors, and the establishment of the temporal association of ARF and sepsis, that is, whether ARF is a complication of sepsis or an exacerbation of a preexisting renal failure in sepsis, are all limitations that may have skewed the results. The dependence on ICD codes and death certificates, which risks distortion or exclusion of ARF and sepsis as a cause of death, must be noted. Additionally, the database does not encompass information on clinical variables that could be used to better report the correlation between ARF and sepsis, such as vital signs, symptoms, laboratory findings, or genetic scans. Details on medical management and other treatment options are not available. Lastly, data concerning socioeconomic determinants, untreated and unreported cases, and racial inequalities in healthcare were also unavailable. Further delineation of the reasons responsible for these disparities is required. These reports are based on data available for the study period (1990–2020) and may not translate results due to recent advances.

## Conclusions

The study demonstrates a significant increase in AAMRs for sepsis and ARF-related deaths among the general population of the United States from 1999 to 2020. The AAMR was consistently higher in men than in women. The highest AAMR were observed among NH black men, the southeastern region of the US, and in metropolitan areas in the specific age strata studied for the adults aged >55 years who had sepsis and acute renal failure-related death. These trends underscore the need for targeted healthcare interventions, preventive strategies, and additional research to address the underlying causes of these disparities. The findings highlighted by this study can prove to be very useful in future healthcare decisions. It can guide efforts required to mitigate the various variables that impact health in adults in the United States. High-risk populations and communities have been identified that can benefit greatly from new policies to better aid their health outcomes. This will contribute to the betterment of overall national health in the United States.

## Acknowledgements

The authors acknowledge the Center for Disease Control and Prevention (CDC) WONDER database for providing open-access mortality data used in this study. No additional assistance or institutional support was received.

## Data Availability

Data are publicly available.

## Research registration unique identifying number (UIN)

Not required because it is a review.

## Peer and provenance statement

Not commissioned, externally peer-reviewed.
